# Trimethylation of Histone 3 lysine 27 (H3K27me3) ChIP-PCR and transcriptional expression data of Ef1-alpha, cyp26A, HoxC10, HoxD10 and HoxD11 in the Xenopus XTC cell line

**DOI:** 10.1016/j.dib.2017.10.056

**Published:** 2017-11-04

**Authors:** Warren Vieira, Hande Sahin, Kaylee Wells, Catherine McCusker

**Affiliations:** Department of Biology, University of Massachusetts Boston, MA 02125, USA

## Abstract

Trimethylation of Histone 3 lysine 27 (H3K27me3) is a chromatin modification that is associated with transcriptional repression (Cao et al., 2002; Sarma et al., 2008; Pengelly et al., 2013) [1], [2], [3]. In this article we performed anti-H3K27me3 Chromosomal Immunoprecipitation (ChIP-PCR), to detect the abundance of H3K27me3 marks on Ef1-alpha, cyp26A, HoxC10, HoxD10 and HoxD11 in the Xenopus XTC cell line. We also performed RT-PCR for these genes to determine whether their expression is detectable in the XTC cell culture. The data we present here are the fold enrichment of Ef1-alpha, cyp26A, HoxC10, HoxD10 and HoxD11 on anti-H3K27me3 ChIP compared to no antibody controls. We also present RT-PCR data on the above listed genes.

**Specifications Table**

**Table:** 

Subject area	Biology
More specific subject area	Epigenetics
Type of data	graph, figure
How data was acquired	Molecular Imager Gel Doc XR system (BioRad)
Data format	Raw and analyzed
Experimental factors	XTC cells grown under normal conditions
Grown Experimental features	Anti-H3K27me3 ChIP-PCR and RT-PCR on samples of XTC cells
Data source location	Boston, Massachusetts
Data accessibility	Data is with this article

**Value of the data**

• The H3K27me3 fold enrichment of Cyp26A, HoxC10, and HoxD10 is greater than that observed with Ef1-alpha, and HoxD11.
• The transcriptional expression of Ef1-alpha is greater than the abundances observed for Cyp26A, HoxC10, and HoxD10, and HoxD11.
• Decreased ChIP enrichment coincides with high transcriptional expression for Ef1-alpha, increased ChIP enrichment coincides with low transcriptional expression for Cyp26A, HoxC10, and HoxD10.
• Little data is currently published on histone modifications in the widely-used XTC cell line.
• This method provides an established ChIP-PCR protocol to use on future experiments in XTC cells.

## 1 Data

**Fig. 1:**
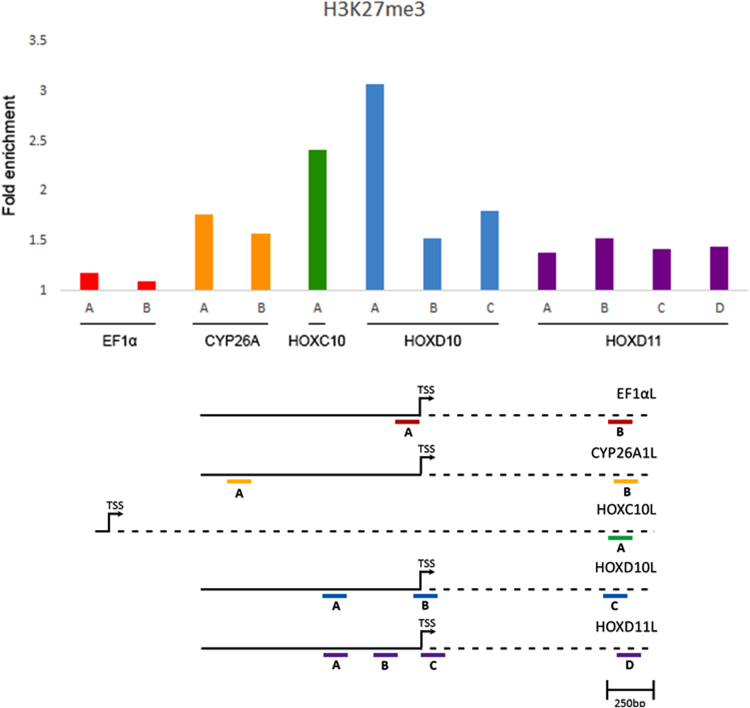
Anti-H3K27me3 ChIP-PCR data for Ef1-alpha, Cyp26A, HoxC10, HoxD10, and HoxD11: (Top panel) Fold enrichment of the abundance of regions of Ef1-alpha, cyp26A, HoxC10, HoxD10 and HoxD11 amplified by different PCR primer sets (A, B, D, or D) for each gene. Fold enrichment was quantified as a ratio of the abundance of each amplimer in the PCR reactions from the ChIP with or without the anti-H3K27me3 antibody. (Lower panel) Schematic representing the location of each primer set that was used for each gene relative to the transcription start site (TSS).

**Fig. 2:**
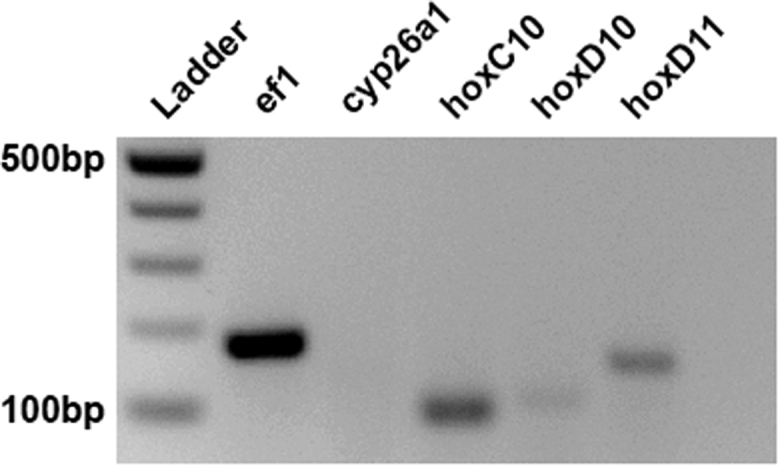
RT-PCR data for Ef1-alpha, cyp26A, HoxC10, HoxD10 and HoxD11 in XTC cells: A semi-quantitative method of analysis was used to determine the relative transcriptional expression of specific genes in the XTC cell line. Regions of the Ef1-alpha, cyp26A, HoxC10, HoxD10 and HoxD11 transcripts were amplified by different PCR primer sets from cDNA libraries generated from unmanipulated XTC cells.

XTC cells are a fibroblastic cell line derived from larval *Xenopus laevis* that is widely used in research laboratories that work with amphibian model systems. Little data is present on the chromatin status of specific genes in this model system, thus new data on the epigenetic status of specific genes in these cells is warranted. Here we provide data for anti-H3K27me3 ChIP-PCR represented as fold enrichment on Ef1-alpha, cyp26A, HoxC10, HoxD10 and HoxD11 in unmanipulated XTC cells (Fig. 1). Since H3K27me3 is a “repressive” chromatin mark that is associated with decreased gene expression [1], [2], [3], we also present raw RT-PCR data for each of the above genes in these cells (Fig. 2).

## 2 Experimental design, materials and methods

### 2.1 XTC tissue culture

XTC cells were cultured with 70% L-15 media (Corning Cellgro) (diluted in molecular grade water) supplemented with Pen/Strep, L-glutamine, sodium pyruvate, and fetal bovine serum (10 U/ml, 2 mM; 0.11 mg/ml, 10%, VWR).

### 2.2 Anti-H3K27me3 ChIP

Anti-H3K27me3 ChIP was performed on XTC cells using a modified version of the protocol described by Edwards and Murray [4]. Modifications were made according ChIP protocols established in *Xenopus Laevis* embryos [5] and mammalian cell lines [6].

A single cell suspension of XTC cells, generated by trypsinization, was washed once with phosphate buffered saline (PBS) and then treated with 1% formaldehyde (diluted in 4 °C PBS) for 45 min on ice, to facilitate DNA cross-linking. The crosslinking reaction was stopped by removing the formaldehyde and adding 0.125 M glycine for 10 min. The cells were washed once in PBS and then twice in isolation buffer (3.75 mM Tris–HCl, pH 8.0, 0.05 mM spermine, 0.125 mM spermidine, 0.5 mM EDTA, 0.5 mM DTT, 20 mM KCl, 0.1 mM phenylmethylsulfonyl fluoride (PMSF)) before being incubated for 10 min on ice in isolation buffer with 0.1% digitonin. The cells were homogenized and the nuclei were collected by centrifugation at 2500 rpm for 5 min. The pelleted nuclei were washed with washing buffer (20 mM HEPES pH8, 20 mM KCl, 0.5 mM EDTA, 0.5 mM DTT, 0.1 mM PMSF) and re-suspended in sonication buffer (washing buffer with 0.3 M NaCl and Halt™ Protease and Phosphatase Inhibitor Cocktail (Thermo Scientific)). Sonication was performed on ice with a Branson Ultrasonics Sonifier™ S-450 for 10 s, with a 20% amplitude and a 20% duty, for 14 cycles. NP-40 was then added to a final percentage of 0.1 and the solution was centrifuged at 14000*g* for 10 min, at 4 °C.

The supernatant was collected; 20 µl was taken as an input sample, being combined with 180 µl ChIP buffer (washing buffer with 0.3 M NaCl, 0.1% NP-40 and 0.1% Tween) and then frozen at −20 °C. The remaining supernatant was pre-cleared with blank Protein A Agarose beads (Roche) overnight, under shaking conditions at 4 °C, and then split in half to generate an experimental and control sample. To the experimental sample, 7.5 µg Rabbit Histone H3K27me3 (H3K27 Trimethyl) Polyclonal Antibody (A-4039-050, Epigentek) attached to beads, pre-blocked in BSA (100 µg), was added and the sample was incubated overnight, with gentle shaking, at 4 °C. The control sample was exposed to blank, pre-blocked beads. The beads were collected by centrifugation at 12000 rpm for 30 s, washed three times with washing buffer with 0.3 M NaCl and 0.1% NP-40, and re-suspend in 200 µl ChiP buffer. The input sample was thawed and processed with the control and experimental samples from this point. Samples were incubated overnight at 65 °C with 200 µg/ml Proteinase K (VWR), with intermittent vortexing during the incubation period. DNA was extracted with phenol/chloroform and precipitated with 0.1 volume 3 M Na Acetate pH 5.2 and 2.5 volumes 100% ethanol at −80 °C overnight. The DNA was ultimately re-suspended in 25 µl TE buffer.

### 2.3 RT-PCR

XTC cells were rinsed with PBS before extracting in TriPure (Roche) and CHCl_3_. The aqueous layer was separated from the organic layer using Phase Lock Gel tubes (VWR), and the aqueous layer was added to 70% ethanol before using a Nucleospin RNA XS kit (Machery-Nagel) to purify the RNA. The Transcriptor First Strand cDNA synthesis Kit (Roche) was used to synthesize cDNA stocks from the purified RNA. PCR was performed on the cDNA stock using Platinum Green Hot Start PCR Master Mix (2×) (Life technologies), and primers for Ef1-alpha, cyp26A, HoxC10, HoxD10 and HoxD11 for 35 cycles.

cyp26a F1: GTTTGCCAAAATCCTCCTCA.

cyp26a R1: TTAGCGGGTAGGTTGTCCAC.

Ef1-alpha F1: TGATGCTCCAGGACACAGAG.

Ef1-alpha R1: CGATCAGCTGCTTAACACCC.

HOXD10 F1: GGCAAACCCCAAGAGTACAA.

HOXD10 R1: TGAGGAGGGATCTGCTGTTT.

HOXD11 F1: TTCTCCAGCAAGTCCTCGTT.

HOXD11 R1: CCCCTGTACTGCCACTTTGT.

HOXC10 F1: CTTATAGCGGATGCCCAAAA.

HOXC10 R1: CATTCTGCTTCGCCATACAA.

PCR products were loaded onto a 2% agarose gel, and the separated bands were documented using the Molecular Imager Gel Doc XR system (BioRad).
